# Prevalence and associated risk factors of scabies and impetigo: A cross-sectional study in Tutume district, Botswana

**DOI:** 10.1371/journal.pntd.0011495

**Published:** 2024-06-03

**Authors:** Leungo Audrey Rainer, Tuduetso Leka Molefi, Sidney Otladisa Kololo, Tshepo Botho Leeme, Mpho Selemogo, Mooketsi Molefi

**Affiliations:** 1 Department of Family Medicine & Public Health, Faculty of Medicine, University of Botswana, Gaborone, Botswana; 2 Disease Control Division, Community Health Services, Ministry of Health, Gaborone, Botswana; University of Sussex, UNITED KINGDOM

## Abstract

**Background:**

The epidemiology of scabies is poorly understood, particularly in regions with high disease burden. This lack of epidemiological data, especially in sub-Saharan Africa, hampers the control and preventative measures. This study is aimed at estimating the prevalence and associated risk factors of scabies and impetigo in the Nata and Sowa catchment areas of Tutume district.

**Methodology:**

A cross-sectional study was conducted in the Tutume District, targeting the settlements of Manxhotae, Malelejwe, Ndutshaa, and Tshwaane. Participants were randomly selected from households in the settlements. Data were collected using questionnaires, and participants were classified as having scabies typical lesions if they met criteria B and or C of International Alliance for the Control of Scabies (IACS) consensus criteria. Statistical significance was set at p<0.05, with a 95% confidence interval for precision.

**Results:**

A total of 429 participants were enrolled across the four settlements. The overall prevalence of scabies was found to be 18.18% (95%CI 14.8–22.1). The highest prevalence of scabies was in Manxhotae at 27.1% (95%CI 21.2–34.0) and Ndutshaa at 23.4% (95%CI 13.4–37.3). Malelejwe and Tshwaane had lower prevalence of 10.4% (95%CI 6.2–16.8) and 3.4% (95%CI 0.8–12.7), respectively. Only five (5) cases of impetigo were identified. Multivariable logistic regression analysis revealed that younger age of 0–4 years, 5–18 years and a household member with an itch were strongly associated with scabies, with adjusted odds ratios (aOR) of 7.9 (95%CI 2.4–25.6) p-value 0.001, 5.7(95%CI 2.7–11.7), p-value 0.001 and 14.3(95%CI 5.3–38.5) p-value 0.001 respectively.

**Conclusion:**

The prevalence of scabies in the Nata catchment area was noted to be high. The risk factors included younger age, a household member with an itch, and less frequent bathing. Prospective studies are needed to explore household disease transmission dynamics and risk factors specific to the youth.

## Author summary

This study was carried out in the Nata catchment area in Tutume district, Botswana. It aimed to assess the burden, risk factors informing effective disease control programs. The survey involved interviews and skin examination by a health worker. Findings revealed a higher prevalence of scabies cases, while impetigo cases were less prevalent. These results highlight the need for community-wide interventions to mitigate the disease’s impact. Identified risk factors include younger age, residing with an individual experiencing itchiness, and infrequent bathing. Overall, this study supports advocating for scabies as a neglected tropical disease.

## Introduction

Scabies was added to the list of Neglected Tropical Diseases (NTDs) in 2017 due to its high burden particularly in areas with limited healthcare access [1]. It is caused by a parasitic mite and often leads to impetigo, a bacterial skin infection. Scabies primarily spreads through direct skin-to-skin contact, and common risk factors include children, the elderly, immunocompromised individuals, overcrowding, and poverty [1–5] Young age seems to be a common risk factor for scabies in high burden settings, which may be due to the fact that children have a maturing immune system and are often in close proximity to each other in schools and even social settings. On the other hand, old age is found to be a risk factor for scabies in low burden countries settings especially in institutionalized settings. In cases of the elderly in nursing homes dementia has a large role to play in the increased risk of scabies [6]. The elderly are likely to experience impaired communication and delayed case diagnosis subsequently resulting in outbreak detection even in low burden countries [6]. Looking into the issue of poverty in low socioeconomic areas, living conditions such as overcrowding, sleeping together, sharing towels and clothing, poor hygiene practices and malnutrition are factors that have been found to increase transmission and predisposition to scabies [3,4]. This necessitates heightened control and prevention interventions. Scabies control efforts by the World Health Organization (WHO) involve mapping disease burden, delivering interventions, and establishing monitoring and evaluation frameworks [1,7,8].

In Botswana, as in many sub-Saharan African settings, the prevalence of scabies is poorly understood, with sporadic cases reported. Lack of epidemiological data hampers the control and preventative measures. Local epidemiological studies therefore are needed to improve our understanding of the disease burden and associated risk factors thereby enabling the development of effective control programs. Previous experiences with other NTDs highlight the importance of mapping to successfully scale up control efforts [1,2]. This study aims to estimate the prevalence of scabies and impetigo, as well as identify associated risk factors in the Nata and Sowa catchment area of Tutume district, Botswana. Such information will contribute to the implementation of appropriate and efficient disease control strategies.

## Methods

### Ethical considerations

Ethical approval was given by the Ministry of Health Human Research and Development committee (reference number: HPRD: 6/13/1). Written permission was obtained from the district health authorities. All participants provided written informed consent. For children within the communities, written consent was obtained from parents or legal guardians and verbal assent was obtained from the children who wished to participate in the survey. All participants diagnosed with scabies or scabies and impetigo were treated with topical permethrin cream alone or in combination with cloxacillin.

### Study design

This was a cross-sectional study performed following a confirmed outbreak. Analytic methods were used to determine possible risk factors associated with scabies.

### Study setting and participants

The study was conducted in the Nata and Sowa catchment area, in Tutume district North-east of Botswana in the May 2022. This catchment area is comprised of a group of villages under Tutume District health management team (DHMT). For operational purposes DHMTs subdivide communities they serve into catchment areas and operate outreach and mobile clinic stops within these. Except for Nata and Sowa, the villages under Nata and Sowa catchment are mostly settlement type and rural with total population ranging from 160 to 700 [9]. The catchment area is predominantly a rural area. The population consists mainly of subsistence farmers and livestock herders. Health care services are provided through a Clinic in Nata, Health post in Manxhotae and mobile stops in the other settlements. Accessibility to health facilities remains a challenge in the settlements due to distance from health facilities. Nata Clinic is a 24-hour clinic and has a full time doctor, nursing practitioners, health care auxiliary and health education assistants while in Manxhotae clinic there are nurses, health education assistant with a doctor who visits only on outreach basis from Nata clinic.

### Inclusion criteria

We included participants living in the selected communities, including primary school going children and willing to participate in the survey. Individuals who did not live in the selected communities, who were unwilling to participate in the study and who were absent at the time of the visit were excluded from the study.

### Sampling

The study was performed during a mass drug administration (MDA) campaign in the district following a confirmed scabies outbreak. The MDA was a recommendation from the outbreak investigation which revealed an estimated prevalence of 19% in a primary school in Manxhotae. As per WHO framework for control of scabies a community prevalence of scabies of > 10% or school prevalence of >15% warrants an MDA [7]. This was the first time MDA done in the district, all households in the selected villages affected by scabies were visited, drug of choice was 5% permethrin as ivermectin is not included in the essential list of medicine for use for scabies in Botswana. For this study villages were purposefully selected then systematic sampling of households was done wherein study participants were randomly selected. Every other household was selected from which we enrolled every second person available. From the 2011 population census projections the estimated population for the respective villages were: Manxhotae 240, Malelejwe 200, Tshwaane 180 and Ndutshaa 160.

### Sample size calculation

The number of participants to be enrolled per site was determined using Cochran’s formula for sample size. Assuming a prevalence of 10% for the community, 7.5% margin of error and a design effect of 2.0 [10] to cater for any clustering that may occur. For each of the sites, the following sample sizes were derived: Settlement Population Sample size Manxhotae 98 Malelejwe 94 Tshwaane 92 Ndutshaa 88.

### Data collection

Prior to data collection community mobilization and sensitization was conducted by district healthcare workers in the communities that were to be surveyed. Tutume DHMT nurses and doctors were trained on clinical diagnosis, clinical management, and public health approach to control of scabies using WHO NTD training slides. Trained health care workers which included doctors and nurse practitioners carried out a standardized skin examination in a private space set aside in the home/house. The field team consisted of 3 teams each lead by a health care worker from Ministry of health supplemented by nurses from Tutume DHMT and local clinics as well as Health Education assistants from the catchment area. Demographic information of the participants as well as clinical information was captured using a questionnaire that was adapted from “A scabies outbreak in the North East Region of Ghana: The necessity for prompt intervention “by Amoako and others [11]. A skin examination of exposed regions of the body was performed on all participants. Scabies was diagnosed guided by the criteria of the International Alliance for the Control of Scabies (IACS) [8,12,13]. Cases meeting criteria B that is clinical scabies and or C, suspected scabies of IACS consensus criteria were considered as rash typical of scabies. Those not diagnosed with scabies and with serious complications were referred for further management in the clinic. IACS criteria has not been validated yet in Botswana, however been used in similar settings like Ghana. Impetigo was diagnosed if a participant had pustules or ulcerative lesions with associated erythema, crusting or pus.

Data was collected on REDCap which is a secure web application. The software is versatile and is able to allow offline data collections, data syncing was then done once internet access was gained. All devices were password protected to ensure confidentiality.

### Statistical analysis

Data captured on REDcap software was transferred to Stata 13 [14] for further management and analysis. Demographic (e.g. sex), and clinical data (e.g. presence of an itch, rash etc) were summarized using frequencies and percentages for categorical variables, while for continuous variables e.g. age, normality tests were run and data summarised appropriately. Prevalence was calculated as the proportion of participants with rash consistent with scabies with or without impetigo out of the total sample. Overall and per site prevalence was estimated. Initially, bivariate analyses were used to determine risk factors indirectly associated with the development of scabies. An inflated p-value of ≤0.2 was used as a criterion for selection of variables into the candidate multivariable regression models. Using stepwise backward elimination method, a parsimonious multivariable regression model was produced. Subsequent to that a Hosmer-Lameshaw goodness-of-fit test (P = 0.08) demonstrated that the model has a good fit with the observed data.

## Results

A total of 429 participants were surveyed in all the selected villages, 188 (43.82%) in Manxotae, 135 (31.47%) in Malelejwe, 59 (13.75%) in Tshwaane, and 47 (10.96%) in Ndutshaa. The characteristics of the participants are displayed in Table 1. 58.2% of the participants were female, while 41.8% were male. The median age is 35 years with an interquartile range of 25 and 56 years.

**Table 1:** Characteristics of study participants and proportion per village.

Characteristics	n (percentage *)
**Gender (n = 412)** ^a^	
Male	172(41.8)
Female	240(58.2)
**Age category(in years) (n = 380)** ^b^	
0–4	20(5.3)
5–18	70(18.4)
19–64	240(63.2)
≥65	50(13.2)
**Village (n = 429)**	
Manxhotae	188(43.8)
Malelejwe	135(31.5)
Tshwaane	59(13.8)
Ndutshaa	47(11.0)

* note that some percentages may not add exactly up to 100% due to rounding-off

^a^ 17 entries had missing data on a gender

^b^ 49 entries had missing data on age

Table 2 shows prevalence of scabies by examination findings stratified per village. The village with the highest prevalence of scabies was Manxhotae, with a prevalence of 27.13%(95%CI 21.22–33.96), followed by Nduutsha with a prevalence of 23.40% (95%CI 13.35–37.73). Malelejwe and Tshwaane had a prevalence of 10.37% (95%CI 6.22–16.80) and 3.39(95%CI 0.84–12.74%) respectively.

**Table 2:** Prevalence of scabies by village using exam findings.

Village	# with scabies (exam)	Population accessed	Prevalence (95%CI)
Manxhotae	51	188	27.1(21.2–34.0)
Malelejwe	14	135	10.4(6.2–16.8)
Tshwaane	2	59	3.4(0.8–12.7)
Ndutshaa	11	47	23.4 (13.4–37.7)
Overall	78	429	18.18(14.8–22.1)

In the diagnosis of scabies by examination of skin lesions, running an unadjusted regression did not yield any statistically significant results (see Table 3).

**Table 3:** Factors associated with the development of scabies.

Variable	cOR (95%CI)	p-value	aOR (95%CI)	p-value
**Age categories**				
0–4	7.0(2.7–18.6)	0.001	7.9(2.4–25.6)	0.001*
5–18	5.4(2.9–10.2)	0.001	5.7(2.7–11.7)	0.001*
19–64	Ref	-	Ref	-
> = 65	1.9(0.8–4.3)		1.8(0.7–4.6)	0.242
**Shared sleeping space+**				
No	Ref	-	Ref	-
Yes	1.7(1.0–2.8)	0.044	1.2(0.6–2.4)	0.537
**Next person has an itch?**				
No	Ref	-	Ref	-
Yes	10.5(4.9–22.4)		14.3(5.3–38.5	0.001*
**Shared bedding**				
No	Ref	-	Ref	-
Yes	2.4(1.4–4.3)	0.002	1.0(0.5–2.2)	0.925
**Shared undergarments**				
No	Ref	-	Ref	-
Yes	2.2(1.2–3.8)	0.008	1.8(0.9–3.8)	0.103

* -statistically significant 95% CI or p-value<0.05 unless otherwise stated

In running an adjusted multivariable binary logistic regression model (see Table 3) only younger age that is those 0–5 years, 6–18 years and those who reported to have had a household contact with an itch had statistically significant odds of having scabies of aOR = 7.9(2.4–25.6) p-value 0.001, aOR = 5.7(2.7–11.7) p-value 0.001 and aOR = 14.3(5.3–38.5) p-value 0.001 respectively.

There were only 5 cases of impetigo, no statistical tests were run to find associations given the low number.

## Discussion

The burden of scabies was found to be highest in Manxhotae with a prevalence of 27.3% while overall prevalence for the Nata and Sowa catchment area was found to be 18.8%. A systematic review which also included grey literature in all regions except North America found that the prevalence of scabies ranged from 0.2% to 71.4% [15]. Most regions had a prevalence of more than 10%, this was in keeping with majority of population based studies in prevalence of scabies [2,4, 5,11,15–20]. The high prevalence of scabies in the Nata and Sowa catchment area which is comparable to otherstudies suggests the need for intensified surveillance and case management to prevent further outbreaks. This high prevalence of the disease is also suggestive of the unappreciation of the burden of scabies in resource-poor communities, and difficult access to the health system. Efficient and effective surveillance mechanisms are needed to properly map and determine the distribution and burden of the disease. In most studies the prevalence of scabies in high burden countries decreased significantly after MDA [2,8,21–23] More than one cycle of MDA may be necessary to control the burden of the disease.

In some studies impetigo was found to be common, particularly in children [2,3,17]. This study only found 5 people with impetigo. The significantly low cases of impetigo found in this study was different from a population-based survey done in Fiji [17] which showed a prevalence of impetigo of 19.6% and several other studies which showed a higher prevalence of co-infection of scabies and impetigo [15–17,19] The co-infection of scabies and impetigo is relatively common, especially in communities with poor hygiene and sanitation. This is because both conditions are highly contagious and tend to affect people living in close proximity to each other and crowded living conditions. According to a study published in the Journal of Global Infectious Disease, the co-infection rate of scabies and impetigo in certain populations ranged from 10–83% and was particularly common in children [2]. In some cases, the presence of scabies may increase the risk of developing impetigo because the intense itching caused by the mites can lead to skin damage and subsequent bacterial infection.

Given that the study was performed during a confirmed scabies outbreak there may have been a degree of selection bias. The population on Nata catchment area also have a higher degree of poverty and overcrowding in households compared to the rest of the country. The villages selected for study are predominantly settlement type of villages. Other challenges also include shortage of water which may have played a role in the high prevalence. The International Alliance for the Control of scabies (IACS) has outlined a case definition for diagnosis of scabies however due to lack of objective diagnostic tests case under-ascertainment and underreporting may be a limiting factor in capturing the true picture of scabies cases on daily basis especially for atypical rash presentations.

Using examination findings to diagnose scabies the unadjusted multivariable regression model did not show any statistically significant results. In the adjusted multivariable binary logistic regression model only younger age less than 18 years and those who reported to be living with someone with an itch in the household emerged statistically significant. The younger age group specifically 5 to 18 years is commonly a school going group and in the Nata settlement area and most schools are boarding. These community findings were in keeping with the findings of the outbreak investigation done in the area which had revealed a higher prevalence of cases in primary schools with Manxhotae primary school as the leading school. Those younger than 5 years have a developing immune system which may increase their susceptibility to transmission. Further studies will need to be done to better understand the variability in the age groups. Due to the weaker association between the other factors and scabies, the effect size diminished in the multivariable model. Although our findings are consistent with a systematic review and meta-analysis carried out in Ethiopia [18] which showed a correlation between the number of members per household and the risk of scabies, the magnitude of association found in our study was not as strong, and therefore the effect diminished in the multivariable model.

This study possesses several strengths and limitations. It stands out as one of the few globally and regionally conducted studies shedding light on the neglected yet debilitating disease’s epidemiology. The analysis conducted in this study identifies established risk factors, such as a higher burden of the disease among the younger population, overcrowding, and its association with low socioeconomic status. However, the study did not explore other potential risk factors, such as the disease’s heightened risk among the HIV population. A post-hoc power analysis, revealed that we had achieved more than 80% power to generate reliable village-level prevalence estimates for Manxhotae and Malelejwe. Therefore, this study may have limitations in terms of statistical power when it comes to Tshwaane and Nduutsha. This limitation could have affected the precision of the estimates for these specific villages. The study only took a sub sample of the households’ members which may have resulted in underestimation of the overall disease prevalence. The main limiting factor was time. We have addressed this by providing a plausible range of prevalence using the 95% confidence interval (CI). This allows us to account for potential variations in scabies prevalence and enhances the robustness of our findings. The authors also acknowledge the relatively imprecise estimates of village-level prevalence due to the low numbers sampled at this level Another possible limitation was gender bias, which may have occurred around participant recruitment; males were less likely to be recruited than females due absence from households.

Furthermore, while our multivariable regression models suggest potential associations between certain exposures and the occurrence of scabies, the lack of temporality between the exposure and outcome variables prevents us from fully establishing the findings. To provide a more comprehensive understanding of the associated factors, future epidemiological studies need to incorporate temporality, ensuring a clear delineation that exposure variables under investigation preceded the outcome.

## Conclusion

The study findings indicate a high overall prevalence of scabies in the Nata and Sowa Catchment area, with the highest prevalence observed in Manxhotae village. Our analysis of examination findings revealed that younger age and a history of someone with an itch in the household were identified as factors strongly associated with scabies. Based on the some of the limitations incurred while doing the study the findings give a snapshot base line of the burden of the scabies and possible factors associated with it. There is therefore need for strengthening surveillance mechanisms and implementation of community-wide interventions to mitigate the disease’s impact.

## Supporting information

S1 Dataset The Tutume district (Nata and Sowa Catchment) data set. (XLSX)
